# Symptom Network Analysis in Patients With Primary Sclerosing Cholangitis

**DOI:** 10.1155/cjgh/5291248

**Published:** 2026-10-14

**Authors:** Melinda Wang, Amy M. Shui, Joanne Hatchett, Stephen J. Rossi, Ruth-Anne Pai, Rachel Gomel, Mary Pressley Vyas, Sarah Curup Callif, Ricky Safer, Jessica Rubin, Christopher Bowlus, Donna M. Evon, Jennifer C. Lai, Michael Li

**Affiliations:** ^1^ Division of Gastroenterology and Hepatology University of California San Francisco California USA berkeley.edu; ^2^ Department of Epidemiology and Biostatistics University of California San Francisco California USA berkeley.edu; ^3^ PSC Partners Seeking a Cure Greenwood Village Colorado USA; ^4^ PSC Partners Seeking a Cure Canada Toronto Ontario Canada; ^5^ University of California Davis Health Sacramento California USA ucdavis.edu; ^6^ Division of Gastroenterology and Hepatology University of North Carolina Chapel Hill North Carolina USA unc.edu

**Keywords:** cluster, fatigue, node, survey, weakness

## Abstract

**Introduction:**

Patients with primary sclerosing cholangitis (PSC) experience numerous symptoms that impact quality of life. We sought to identify PSC‐specific symptom clusters and relationships between symptoms to better understand the lived experience of PSC patients.

**Methods:**

Presence and severity of 16 symptoms were identified from the international “Our Voices” survey collected by PSC Partners Seeking A Cure in 2020. LASSO‐regularized partial correlation network analysis was performed using Spearman correlations. The primary analysis utilized symptoms reported within the past 6 months of survey completion, with subgroup analyses conducted on patients with and without inflammatory bowel disease (IBD). Expected influence was computed to quantify the importance of each measure within networks. The Spinglass algorithm was employed to identify communities within networks.

**Results:**

Among 970 total participants, 54% were women, and 70% had IBD. Four dominant communities were identified across networks, including global symptoms, psychological symptoms, gastrointestinal symptoms, and cholestatic‐related symptoms. Of the 16 symptoms included in network analyses, fatigue and weakness had the most positive influence across multiple networks and across different communities within each network.

**Conclusions:**

The complex relationship between co‐occurring symptoms in PSC organizes into four dominant symptom clusters. FTG and WEA emerged as strongly influential symptoms that consistently were associated with other symptoms. Our analyses emphasize the complex, co‐occurring nature of PSC symptoms, identify highly influential symptoms, and highlight the need to develop patient‐reported outcome symptom measures to assess changes in these key symptoms in clinical trials and clinical practice.

## 1. Introduction

Primary sclerosing cholangitis (PSC) is a rare, chronic cholestatic liver disease characterized by inflammation and stricturing of bile ducts, often leading to the development of end‐stage liver disease [1, 2]. Approximately 70%–80% of patients with PSC have inflammatory bowel disease (IBD) [3]. Half of people with PSC are asymptomatic and present only with elevated alkaline phosphatase at diagnosis [4]. Those with symptoms may present with pruritus (PRU) or abdominal pain (ABD). Other symptoms such as fatigue (FTG) and sleep disturbance can occur at any stage of disease [1]. Health‐related quality of life (HRQoL) studies highlight the burden of anxiety (ANX) and depression (DEP) elicited by disease progression, uncertainty, lack of effective treatment options, and risk of malignancies [4–6]. Symptoms such as pain, PRU, and FTG have also been associated with poor HRQoL [7, 8]. While PSC‐related symptoms clearly impact HRQoL, it remains unclear how PSC symptoms relate to one another.

The Externally‐Led Patient‐Focused Drug Development (EL‐PFDD) program is an FDA initiative to ensure that patient experiences, needs, and priorities are captured and meaningfully incorporated into drug development and evaluation [9, 10]. Data from EL‐PFDD initiatives across numerous disciplines and diseases have been used to highlight the patient voice, identify unmet clinical needs, and develop patient‐reported outcome measures (PROMs) [11–13]. An EL‐PFDD Forum for patients with PSC was conducted in October 2020 and generated data through a patient survey to better characterize the patients with PSC experience and therapeutic priorities [14]. Network analysis is a statistical method in systems biology that has been harnessed to visualize and understand the interrelationship of symptoms in disease processes in order to inform treatment options [15–18]. Given the high symptom burden associated with PSC, we sought to use network analysis to identify PSC‐specific symptom clusters and elucidate relationships between symptoms.

## 2. Materials and Methods

### 2.1. Study Population

PSC Partners developed the 40‐question “Our Voices” survey as part of the PSC EL‐PFDD Forum, which was distributed to patients enrolled in the PSC Partners Patient Registry starting in July 2020. The registry is patient‐driven, web‐based, and de‐identified in accordance with the NIH standards for privacy and confidentiality [19]. The survey was also made available through an external website to patients not enrolled in the registry. Adult patients 18 years or older who completed the survey before the EL‐PFDD Forum were included in this study.

The *Our Voices* survey included four main symptom domains. Patients were asked, “When your PSC symptoms were AT THEIR WORST, how much did the following symptom IMPACT how you felt and functioned?” and responded using a 3‐point ordinal scale (0 = *Never*, 1 = *Minor impact*, 2 = *Major impact*). Patients were asked, “IN THE PAST 6 MONTHS, what IMPACT are the following symptoms having on how you feel and function?” with the same 3‐point ordinal scale. Patients were additionally asked, “CURRENTLY, what IMPACT is the following PSC symptom having on how you feel and function?” using the same 3‐point ordinal scale. The analyses for this research were approved by the Institutional Review Board at the University of California, San Francisco.

### 2.2. Summary Statistics

Patient characteristics, including age, gender, race/ethnicity, cirrhosis, IBD, transplant status, timing of symptoms, and medications, were summarized. Participants were divided into patients with and without IBD and compared using chi‐square tests. Frequencies of symptoms within 6 months, at the time of survey, and at their worst were also summarized.

### 2.3. Network Analysis

To examine the relationships between several measures simultaneously in our study cohort, the *R* package “qgraph” was utilized to perform a Least Absolute Shrinkage and Selection Operator (LASSO)‐regularized partial correlation network analysis using the nonparametric Spearman correlation matrix method on 16 PSC‐related symptoms [9–12]. The Fruchterman–Reingold algorithm was used to visualize the networks; green edges in each network graph represent positive associations, red edges represent negative associations, and the thickness of edges represents the strength of associations. Five separate network analyses were performed of 16 patient‐reported symptoms. Network 1 (primary analysis) included symptoms reported in the past 6 months (*n* = 764). Network 2 included current symptoms at time of survey completion (*n* = 758). Network 3 included symptoms at their worst (*n* = 958). Network 4 included symptoms reported in the past 6 months among patients with concurrent IBD (*n* = 530), while Network 5 included symptoms reported in the past 6 months among patients without IBD (*n* = 234). Patients who received a liver transplant were excluded from all networks except for Network 3, which assessed symptoms at their worst.

One‐step expected influence (EI1) and two‐step expected influence (EI2) were computed to quantify the importance of each measure within the network. Expected influence is the degree that nodes, represented as symptoms, impact the whole network, accounting for how well connected a node is and how many links their connections have [12]. Expected influence incorporates aspects of node strength (number of links held by each node), betweenness (number of times a node lies on the shortest path between nodes), and closeness (a measure of the shortest path between nodes). EI1 and EI2 account for the presence of negative edges, where the highest z‐score represents the most positive influence, and the lowest z‐score represents the least positive influence. EI1 only incorporates information about the influence of a node’s immediate neighbors. EI2 accounts for both the immediate influence of a node within the network and the secondary influences on the network through its neighbors. A node’s EI2 is its EI1 plus the sum of the EI1 values of the remaining nodes in the network.

The Spinglass algorithm was used to determine communities within each network. Bridge centrality, which measures the importance of nodes in connecting communities, was assessed using EI1 and EI2. Spearman correlation coefficients were used to determine which nodes from each community were strongly correlated with the most influential bridging nodes. Network stability analyses were performed on the primary analysis network. Bridge centrality analysis was performed on all networks. Nonparametric bootstrapping (1000 samples) was used to estimate stability of edge weights. Case‐dropping subset bootstrap samples (*n* = 1000) were used to estimate the stability of centrality indices, with a correlation stability (CS) coefficient for correlation values ≥ 0.7 (CS (*r* = 0.7)) [18]. The CS coefficient quantifies the maximum proportion of cases that can be dropped to retain, with 95% certainty, a correlation between our sample’s indices and our case‐dropped bootstraps’ indices of 0.7 or higher. Epskamp et al. suggest that the CS coefficient should not be below 0.25 and preferably above 0.5 [18].

Network analyses were performed using the “qgraph,” “IsingFit,” “bootnet,” and “networktools” packages and the “glasso” algorithm in *R* (Version 4.2.1, Vienna, Austria). Data preparation and other analyses were performed using SAS (Version 9.4, Cary, NC).

## 3. Results

Of 970 participants, 10% of patients were 18–25 years old, 30% were 26–39 years old, 40% were 40–59 years old, and 20% were 60–79 years old. Most patients were White (96%), 54% were women, 70% had IBD, and 18% reported a diagnosis of cirrhosis. Most patients (55%) developed symptoms prior to PSC diagnosis, and 88% ultimately developed PSC‐related symptoms. Patient characteristics and treatment details for the cohort stratified by IBD status are provided in Table 1. A summary of the PSC‐related symptoms assessed in the survey and their impact on quality of life is provided in Supporting Table 1, and illustrative patient quotes for various symptoms are included in Supporting Table 2.

**TABLE 1:** Sociodemographic and clinical characteristics of patients with PSC–IBD versus no IBD.

Characteristic	Frequency *N* = 970	Without IBD *N* = 289 (30)	With IBD *N* = 681 (70)	*p* value
Age				< 0.001
18–25 years old	99 (10)	16 (6)	83 (12)	
26–39 years old	289 (30)	73 (25)	216 (32)	
40–59 years old	385 (40)	115 (40)	270 (40)	
60–79 years old	197 (20)	85 (30)	112 (16)	
Age at diagnosis				< 0.001
Under 5 years old	6 (0.6)	0 (0)	6 (0.9)	
5–12 years old	24 (3)	5 (2)	19 (3)	
13–17 years old	64 (7)	10 (3)	54 (8)	
18–25 years old	187 (19)	36 (13)	151 (22)	
26–39 years old	335 (35)	96 (33)	239 (35)	
40–50 years old	287 (30)	106 (27)	181 (17)	
60–79 years old	65 (7)	34 (12)	31 (5)	
≥ 80 years old	1 (0.1)	1 (0.4)	0 (0)	
Gender				0.08
Male	439 (45)	115 (40)	324 (48)	
Female	524 (54)	172 (60)	352 (52)	
Other	4 (0.4)	1 (0.4)	3 (0.4)	
Race				0.36
White	902 (96)	260 (93)	642 (96)	
Black	17 (2)	7 (2.5)	10 (2)	
Asian	17 (2)	8 (2.9)	9 (1)	
Mixed	12 (1)	4 (1.4)	8 (1)	
Indigenous	2 (0.2)	1 (0.4)	1 (0.2)	
Ethnicity				0.08
Hispanic Latino	25 (3)	12 (4)	13 (2)	
Non‐Hispanic Latino	68 (7)	19 (7)	49 (7)	
Non‐Hispanic Non‐Latino	801 (85)	233 (82)	568 (86)	
Prefer not to answer	50 (5)	20 (7)	30 (5)	
AIH overlap	94 (10)	35 (12)	59 (9)	0.10
Cirrhosis	175 (18)	56 (19)	119 (17)	0.48
Cholangiocarcinoma	21 (2)	7 (2)	14 (2)	0.72
Clinical trial participation				0.94
Never participated	722 (74)	213 (75)	509 (75)	
Recruited	139 (14)	43 (15)	96 (14)	
Current participant	18 (2)	6 (2)	12 (2)	
Past participant	87 (9)	24 (8)	63 (9)	
Listed for transplant	41 (4)	9 (3)	32 (5)	0.26
DDLT	142 (15)	36 (13)	106 (16)	0.21
LDLT	62 (6)	15 (5)	47 (7)	0.32
PSC recurrence (includes not transplanted)	156 (16)	71 (25)	85 (13)	< 0.001
Symptoms				0.02
Never	115 (12)	31 (11)	84 (12)	
Before diagnosis	529 (55)	178 (62)	351 (52)	
Near diagnosis	159 (16)	42 (15)	117 (17)	
After diagnosis	166 (17)	37 (13)	129 (19)	
PSC medications				
Ursodiol	503 (52)	168 (58)	335 (39)	0.01
Steroids	114 (12)	34 (12)	80 (12)	1.00
Other chronic antibiotics	68 (7)	25 (9)	43 (6)	0.19
Vancomycin	63 (6)	7 (2)	56 (8)	0.001
Trial medications	14 (1)	5 (2)	9 (1)	0.63
Other medications	138 (14)	54 (19)	84 (12)	0.01
Symptom medications				
Pain medications	138 (14)	50 (17)	88 (13)	0.07
Pruritus medications	180 (19)	58 (20)	122 (18)	0.43
Nausea medications	142 (15)	38 (13)	104 (15)	0.39
Antidepressants	145 (15)	47 (16)	98 (14)	0.45
Fatigue medications	25 (3)	8 (3)	18 (3)	0.91
Trial medications	9 (1)	2 (0.7)	7 (1)	0.62
Other medications	71 (7)	24 (8)	47 (7)	0.44
IBD medications	N/A		N/A	N/A
Aspirin		269 (30)		
Steroids		68 (10)		
Immunosuppressants		96 (14)		
Biologics		142 (21)		
Tofacitinib		7 (1)		
Trial medications		0 (0)		
Other medications		76 (11)		
Liver disease medications				
AIH overlap medications	74 (8)	23 (8)	51 (7)	0.80
Post‐transplant medications	173 (18)	41 (14)	132 (19)	0.05
Encephalopathy medications	40 (4)	14 (5)	26 (4)	0.46
Probiotics	133 (14)	41 (14)	92 (14)	0.78
Trial medications	10 (1)	4 (2)	6 (1)	0.48
Other medications	53 (5)	16 (6)	37 (5)	0.95

### 3.1. Networks

Figure 1A shows the relationships between the nodes in Network 1. The network was made up of 16 nodes representing symptoms reported in the past 6 months, and network density was 88 out of 120 possible connections observed, consisting of 73% of all possible connections. Based on EI1 and EI2, FTG had the most positive influence in this network, followed by weakness (WEA), while jaundice (JAU) had the least positive influence, followed by encephalopathy (ENC) (Supporting Figure 1A). The network had strong edge and expected influence stability coefficients (both 0.75) (Supporting Figure 1B). Four communities were determined by the Spinglass algorithm (Figure 1B). The four communities included “global symptoms” (red, Community 1), “psychological symptoms” (green, Community 2), “gastrointestinal symptoms” (blue, Community 3), and “cholestatic‐related symptoms” (purple, Community 4). Insomnia (INS) had the most positive bridge EI1 and EI2, followed by FTG, while joint pain (JNT) and ENC had the least positive EI1 and EI2 (Table 2). Items most strongly correlated with INS from other communities included FTG within community 1 (*r* = 0.52), DEP within community 2 (*r* = −0.42), and nausea/vomiting (VOM) within community 3 (*r* = 0.41). Items most strongly correlated with FTG from other communities included ABD within community 3 (*r* = 0.50), INS within community 4 (*r* = 0.52), and DEP within community 2 (*r* = 0.42). The network had an adequate bridge expected influence stability coefficient (0.67).

**FIGURE 1:**
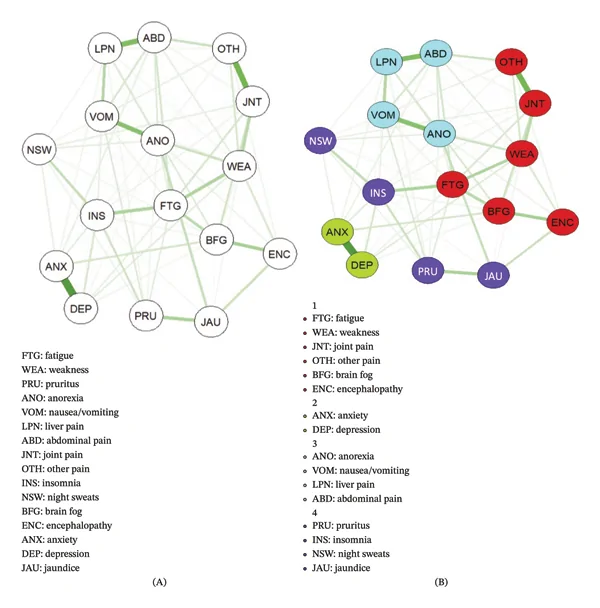
(A) LASSO‐regularized partial correlation network of 16 symptoms reported in the past 6 months (*n* = 764). (B) LASSO‐regularized partial correlation network of 16 symptoms reported in the past 6 months (*n* = 764) using communities determined by the Spinglass algorithm.

**TABLE 2:** Symptoms with the most and least positive bridge influence in each network.

Network	Category	One‐step expected influence (EI1)	Two‐step expected influence (EI2)
1: symptoms reported in the past 6 months (*n* = 764)	Most positive bridge	INS: insomnia	INS: insomnia
		FTG: fatigue	FTG: fatigue
	Least positive bridge	JNT: joint pain	ENC: encephalopathy
		ENC: encephalopathy	JNT: joint pain

2: current symptoms (*n* = 758)	Most positive bridge	WEA: weakness	WEA: weakness
		FTG: fatigue	FTG: fatigue
	Least positive bridge	PRU: pruritus	PRU: pruritus
		JAU: jaundice	JAU: jaundice

3: symptoms reported at their worst (*n* = 958)	Most positive bridge	WEA: weakness	WEA: weakness
		FTG: fatigue	FTG: fatigue
	Least positive bridge	PRU: pruritus	PRU: pruritus
		VOM: vomiting	ABD: abdominal pain

4: symptoms reported in the past 6 months among patients with IBD (*n* = 530)	Most positive bridge	FTG: fatigue	BFG: brain fog
		INS: insomnia	FTG: fatigue
	Least positive bridge	JNT: joint pain	JNT: joint pain
		DEP: depression	JAU: jaundice

5: symptoms reported in the past 6 months among patients without IBD (*n* = 234)	Most positive bridge	FTG: fatigue	FTG: fatigue
		ANO: anorexia	INS: insomnia
	Least positive bridge	ENC: encephalopathy	ENC: encephalopathy
		JAU: jaundice	JAU: jaundice

Figure 2A shows Network 2, consisting of 16 nodes representing current symptoms at time of survey completion. Network density was 83 out of 120 possible connections observed, consisting of 69% of all possible connections. Based on EI2, WEA had the most positive influence in this network, followed by FTG, while JAU had the least positive influence, followed by PRU (Table 2, Supporting Figure 2). Four communities were identified using the Spinglass algorithm (Figure 2B). WEA had the most positive bridge EI2, followed by FTG, while PRU had the least positive EI2, followed by JAU. Items most strongly correlated with WEA from other communities included DEP within community 2 (*r* = 0.40), VOM within community 3 (*r* = 0.54), and FTG within community 4 (*r* = 0.61). Items most strongly correlated with FTG from other communities included WEA within community 1 (*r* = 0.61), DEP within community 2 (*r* = 0.43), and ABD within community 3 (*r* = 0.49).

**FIGURE 2:**
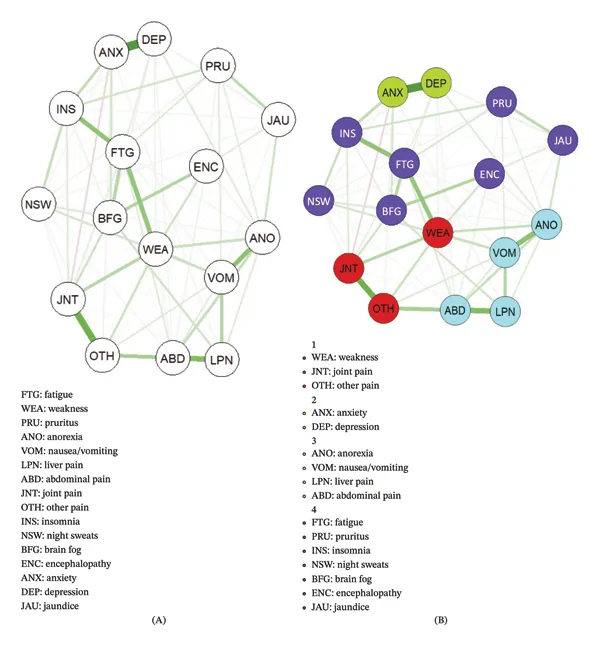
(A) LASSO‐regularized partial correlation network of 16 symptoms reported currently (*n* = 758). (B) LASSO‐regularized partial correlation network of 16 symptoms reported currently (*n* = 758) using communities determined by the Spinglass algorithm.

Figure 3A shows Network 3, consisting of 16 nodes representing symptoms reported at their worst. Network density was 81 out of 120 possible connections observed, consisting of 68% of all possible connections. Based on EI1 and EI2, WEA had the most positive influence in this network, followed by FTG, while ENC had the least positive influence, followed by PRU (Table 2, Supporting Figure 3). Six communities were identified by Spinglass algorithm (Figure 3B). WEA had the most positive bridge EI1 and EI2, followed by FTG. PRU had the least positive EI1 and EI2. Items most strongly correlated with WEA from other communities included DEP within community 1 (*r* = 0.34), brain fog (BFG) within community 2 (*r* = 0.40), ANO within community 4 (*r* = 0.50), JAU within community 5 (*r* = 0.41), and other pain (OTH) within community 6 (*r* = 0.48). Items most strongly correlated with FTG from other communities included DEP within community 1 (*r* = 0.37), BFG within community 2 (*r* = 0.45), ANO within community 4 (*r* = 0.49), INS within community 5 (*r* = 0.42), and OTH within community 6 (*r* = 0.39).

**FIGURE 3:**
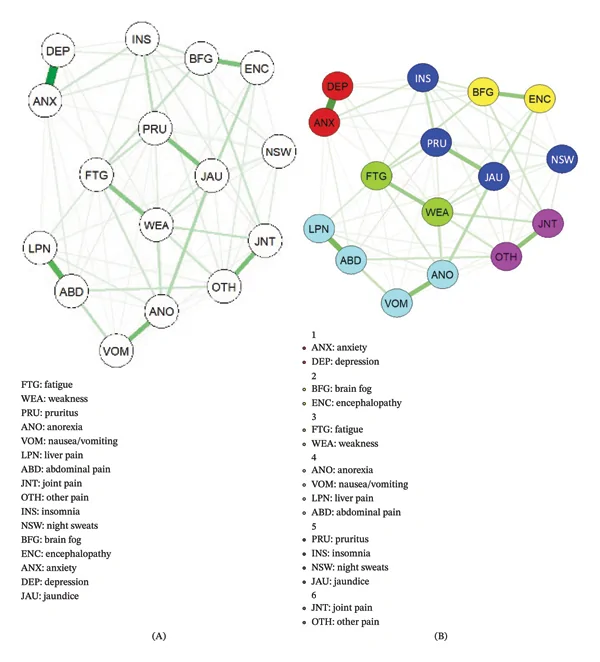
(A) LASSO‐regularized partial correlation network of 16 symptoms reported at their worst (*n* = 958). (B) LASSO‐regularized partial correlation network of 16 symptoms reported at their worst (*n* = 958) using communities determined by the Spinglass algorithm.

Figure 4A shows Network 4, consisting of 16 nodes representing symptoms reported in the past 6 months among patients with concurrent IBD. Network density was 82 out of 120 possible connections observed, consisting of 68% of all possible connections. Based on EI1 and EI2, FTG also had the most positive influence in this network, followed by WEA, while JAU had the least positive influence, followed by PRU (Table 2, Supporting Figure 4). Six communities were identified by the Spinglass algorithm (Figure 4B). FTG followed by INS and BFG had the most positive EI1, while BFG followed by FTG and INS had the most positive EI2. JNT had the least positive EI1 and EI2. Items most strongly correlated with FTG from other communities included BFG within community 2 (*r* = 0.52), INS within community 3 (0.51), ABD within community 4 (0.52), DEP within community 5 (*r* = 0.41), and PRU within community 6 (*r* = 0.36). Items most strongly correlated with BFG from other communities included FTG within community 1 (*r* = 0.52), INS within community 3 (*r* = 0.40), liver pain (LPN) within community 4 (*r* = 0.45), ANX within community 5 (*r* = 0.41), and PRU within community 6 (*r* = 0.23). Items most strongly correlated with INS from other communities included FTG within community 1 (*r* = 0.51), BFG within community 2 (*r* = 0.40), VOM within community 4 (*r* = 0.43), DEP within community 5 (*r* = 0.39), and PRU within community 6 (*r* = 0.34).

**FIGURE 4:**
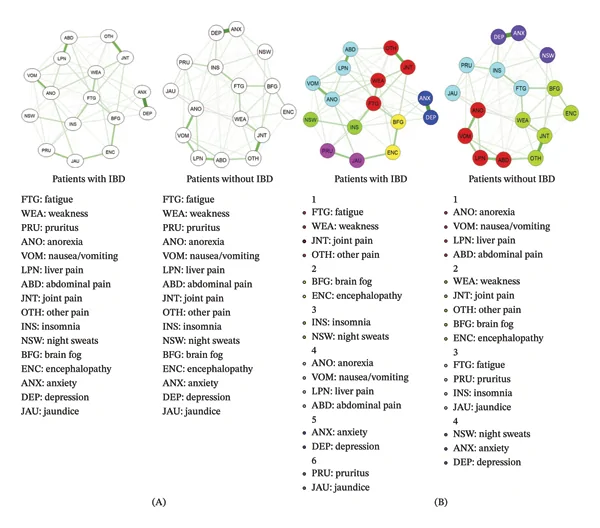
(A) LASSO‐regularized partial correlation network of 16 symptoms reported in the past 6 months among patients with IBD (*n* = 530) and among patients without IBD (*n* = 234). (B) LASSO‐regularized partial correlation network of 16 symptoms reported in the past 6 months among patients with IBD (*n* = 530) versus patients without IBD (*n* = 234) communities determined by the Spinglass algorithm.

Figure 4B shows Network 5, consisting of 16 nodes representing symptoms reported in the past 6 months among patients without IBD. Network density was 78 out of 120 possible connections observed, consisting of 65% of all possible connections. Based on EI1 and EI2, ANO also had the most positive influence in this network, followed by FTG from EI1 and ABD from EI2, while JAU had the least positive influence, followed by ENC (Table 2, Supporting Figure 5). Four communities were identified by the Spinglass algorithm (Figure 4B). FTG followed by ANO and INS had the most positive EI1, while FTG followed by INS and ANO had the most positive EI2. ENC had the least positive EI1 and EI2. Items most strongly correlated with FTG from other communities included ANO within community 1 (*r* = 0.47), WEA within community 2 (*r* = 0.53), and DEP within community 4 (0.43). Items most strongly correlated with INS from other communities included ANO within community 1 (*r* = 0.47), WEA within community 2 (*r* = −0.43), and DEP within community 4 (*r* = 0.50). Items most strongly correlated with ANO from other communities included WEA within community 2 (*r* = 0.47), FTG within community 3 (*r* = 0.47), and DEP within community 4 (*r* = 0.44).

### 3.2. Findings Conserved Across Networks

Supporting Table 3 presents a summary of the symptoms with the most and least influence in each network. Based on EI1 and EI2, FTG and WEA had the most positive influence in all networks, except for Network 5 (symptoms among patients without IBD), in which ANO had the most positive influence, followed by FTG from EI1 and ABD from EI2. FTG was also one of the strongest symptoms in terms of bridge centrality, connecting communities within each of the five networks (Supporting Table 4). Symptoms with the lowest positive influence in each network included JAU, ENC, NSW, and PRU.

Supporting Table 4 displays symptom communities identified within each of the networks. While nuances of the communities differed slightly across each network, 4 dominant symptom clusters/communities arose: global symptoms, cholestatic‐related symptoms, psychological symptoms, and gastrointestinal symptoms; these remained relatively conserved across network analyses.

## 4. Discussion

While prior studies have described symptoms commonly associated with PSC [4, 13, 14], how PSC symptoms cluster and relate to one another was unknown. Understanding the complex interrelationship between symptoms in PSC offers an opportunity to identify highly influential symptoms, which, if improved with treatment, may synergistically ameliorate symptoms and help patients feel and function better.

Among 16 symptoms, FTG and WEA consistently had the most positive influence across the various networks. These data suggest that FTG and WEA are dominant symptoms that need to be assessed as part of clinical trial endpoints for novel PSC therapies and addressed in clinical practice with patient‐centered symptom management. Positive bridge symptoms between communities should also be assessed as clinical trial endpoints, as they affect multiple symptom clusters. Our data identified INS, FTG, and WEA as important positive bridge symptoms across all symptom networks. On the other hand, PRU was consistently identified as the least influential symptom across multiple networks. Notably, this does not mean that PRU is not a highly impactful symptom in terms of quality of life but instead indicates that PRU appears to be less associated or interconnected with global PSC symptoms compared to other symptoms and therefore should be treated as a separate, distinct entity separate from other symptom clusters in clinical trial design. PRU is known to be a highly impactful symptom, as it is the predominant symptom associated with willingness to participate in clinical trials [14, 19]. Furthermore, PRU (19%) was the most common symptom for which patients were taking medications. Interestingly, despite FTG consistently being the most influential symptom identified in our study and being an independent predictor of poor quality of life among patients with PSC [7], only 3% of patients reported taking medications to specifically treat FTG. This may be because no clear therapies for PSC‐related FTG exist, while several evidence‐based medications for cholestatic PRU are available [20]. Whether treatment of these symptoms may synergistically ameliorate other symptoms requires further research. However, bridge and influential symptoms are potential therapeutic targets that may have a significant impact on improving overall quality of life among patients with PSC if treated. Although global WEA is a challenging symptom without clear anatomical specificity to measure, bridge, and influential symptoms such as FTG and INS have validated PROMs in a variety of chronic diseases and are ripe candidate symptoms for PROM development in clinical trials for patients with PSC [21, 22].

Challenges in symptom research among PSC patients include the strong association between IBD and PSC. We hypothesized that patients with both PSC and IBD may have different symptom clusters than patients with PSC without IBD. We found that symptom networks between these two patient populations were similar, although with some interesting differences observed. Anorexia was the most influential symptom among patients without IBD while FTG was the most influential symptom among patients with IBD. FTG remained one of the most positive bridge symptoms in both patient populations. In our study, 70% of patients had IBD and were therefore the dominant phenotype, though PSC symptoms including PRU, FTG, ABD, and fever may not differ in patients with and without IBD [14].

This study has several limitations. First, the network analyses were dependent on the symptoms listed in the *Our Voices* survey and the specific sociodemographic characteristics of the survey participants. Further research is needed to assess the generalizability of our findings. This cohort of PSC patients had a higher female prevalence than the greater PSC population; however, on sensitivity analysis, FTG and WEA remained among the most influential symptoms in both female and male patients. Symptom survey data were also patient self‐reported. Additionally, patients’ experiences of symptoms like FTG and WEA are complex and may not be fully characterized through survey questions simply asking about the presence/absence of symptoms, even considering the ordinal data that were available in this study. FTG and WEA being highly influential symptoms across multiple networks and the complex nature of the patient experience of FTG and WEA highlight the need for in‐depth qualitative studies to better understand the experience of complex symptoms to develop reliable and valid PROMs specific to multifaceted PSC symptoms. Development of PROMs for highly influential symptoms like FTG and WEA is needed to enable assessment of these symptoms as endpoints in clinical trials and clinical practice.

## 5. Conclusions

This study uses a novel network analysis approach to characterize the complex interrelationship among symptoms experienced by patients with PSC using data derived directly from the lived experiences of patients with PSC, which often cannot be elucidated through clinical records alone. We found expected correlations between symptoms such as ANX and DEP, JAU and PRU, and anorexia, VOM, LPN, and ABD. The network analyses also highlighted FTG as a highly influential symptom across networks. Our data can be used to provide a patient‐centered approach to symptom management and emphasize the need to develop validated PSC‐specific PROMs for highly influential symptoms, which can be used to accurately capture change in specific PSC symptoms associated with novel PSC therapies.

## Funding

This study was supported by UCSF Liver Center P30DK026743 (Lai), PSC Partners Young Investigator Award (Li), and NIH T32DK060414 (MW).

## Conflicts of Interest

M.L. receives research funding from P.S.C. Partners Seeking a Cure (paid directly to UCSF). D.E. receives research funding from P.S.C. Partners Seeking a Cure (paid directly to UNC). C.L.B. receives research grants from Calliditas Therapeutics, ChemoMab, COUR Pharmaceuticals, Cymabay Therapeutics, Gilead Sciences, G.S.K. Pharmaceuticals, Hanmi Pharmaceuticals, Intercept Pharmaceuticals, Ipsen Bioscience, Mirum Pharmaceuticals, Novartis, Novo Nordisk, Pliant Therapeutics, Viking Therapeutics, and Zydus Therapeutics. C.L.B. also consults for Alnylam, ChemoMab, Gilead Sciences, Ipsen Bioscience, and NGM Bio. The other authors declare no conflicts of interest.

## Supporting Information

Additional supporting information can be found online in the Supporting Information section.

## Supporting information


**Supporting Information 1** Supporting Figure 1A. One‐step and two‐step expected influence z‐scores for the LASSO‐regularized partial correlation network of 16 symptoms reported in the past 6 months. Supporting Figure 1B. Edge weights stability plot of bootstrapped confidence intervals around the estimated edge weights for the LASSO‐regularized partial correlation network of 16 symptoms reported in the past 6 months. Supporting Figure 2. Expected influence z‐scores for the LASSO‐regularized partial correlation network of 16 symptoms reported currently. Supporting Figure 3. Expected influence z‐scores for the LASSO‐regularized partial correlation network of 16 symptoms reported at their worst. Supporting Figure 4. One‐step and two‐step expected influence z‐scores for the LASSO‐regularized partial correlation network of 16 symptoms reported in the past 6 months among patients with IBD. Supporting Figure 5. One‐step and two‐step expected influence z‐scores for the LASSO‐regularized partial correlation network of 16 symptoms reported in the past 6 months among patients without IBD.

**Supporting Information 2** Supporting Table 1. Impact of symptoms on how patients with PSC feel and function. Supporting Table 2. Illustrative quotations of patients with PSC and their symptoms. Supporting Table 3. Symptoms with most and least positive influence in each network. Supporting Table 4. Community summaries across network analyses by symptom.

## Data Availability

The data supporting the findings of this study are not openly available due to sensitivity and are available from the corresponding author upon reasonable request.
