# Supplementary material for: Symptom Network Analysis in Patients With Primary Sclerosing Cholangitis
Source: Can J Gastroenterol Hepatol. 2026 Oct 14;2026:5291248. doi: 10.1155/cjgh/5291248 (PMC13662822; doi:10.1155/cjgh/5291248)

Supplementary Figure 1A. One-step and two-step expected influence z-scores for LASSO regularized partial correlation network of 16 symptoms reported in the past 6 months (n=764)

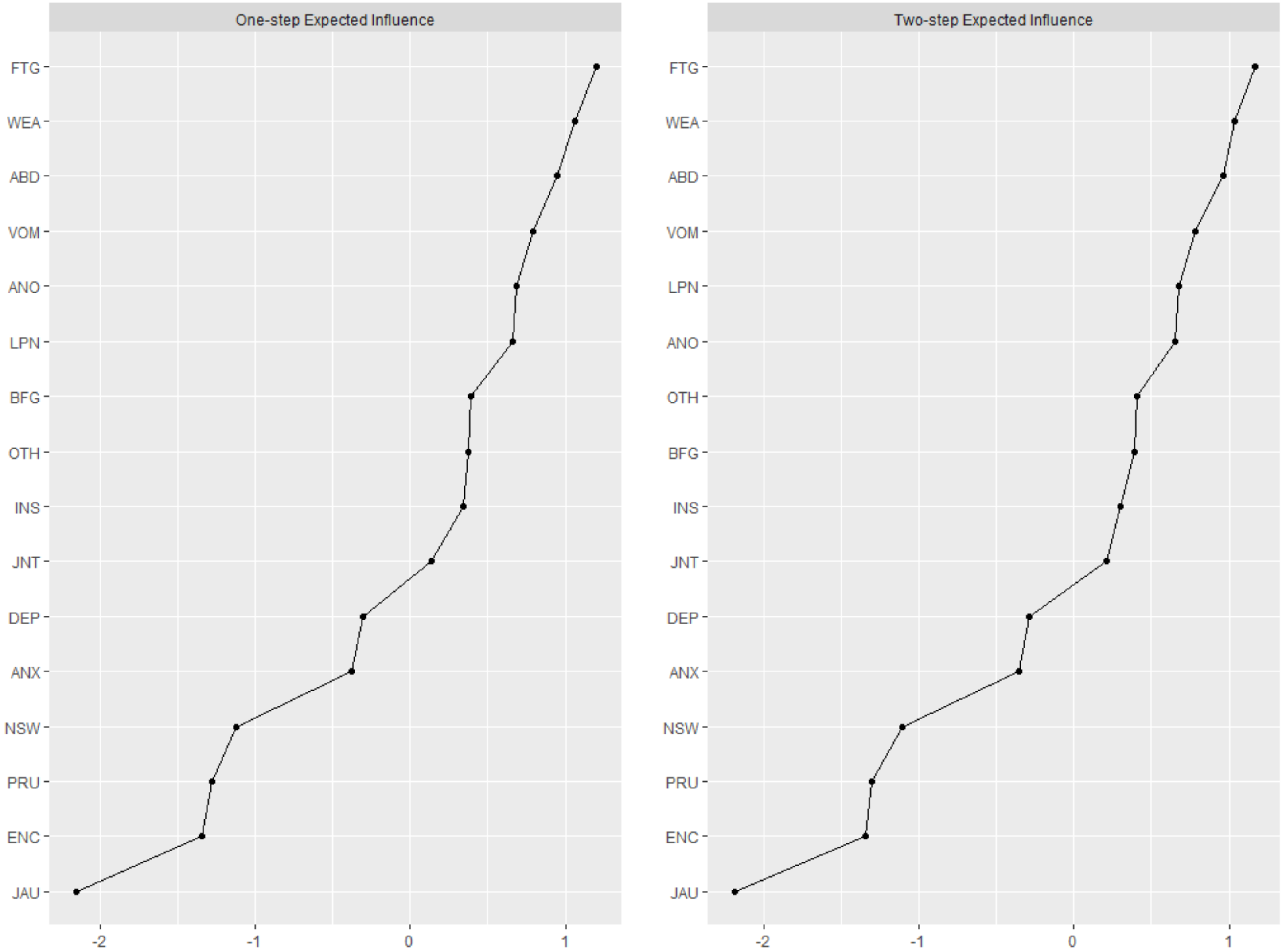

Supplementary Figure 1B. Edge weights stability plot of bootstrapped confidence intervals around the estimated edge weights for LASSO regularized partial correlation network of 16 symptoms reported in the past 6 months (n=764)

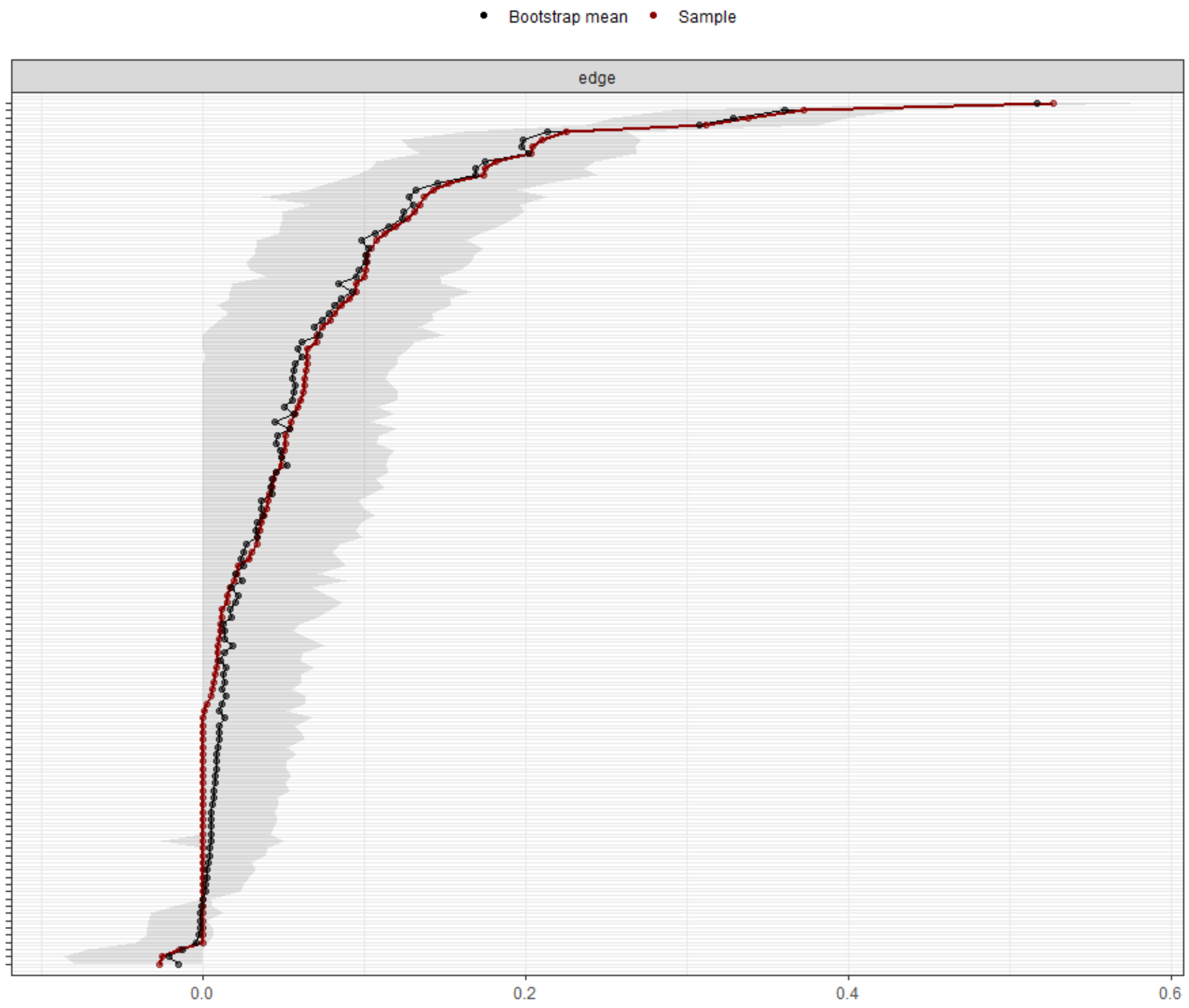

Supplementary Figure 2. Expected influence z-scores for LASSO regularized partial correlation network of 16 symptoms reported currently (n=758)

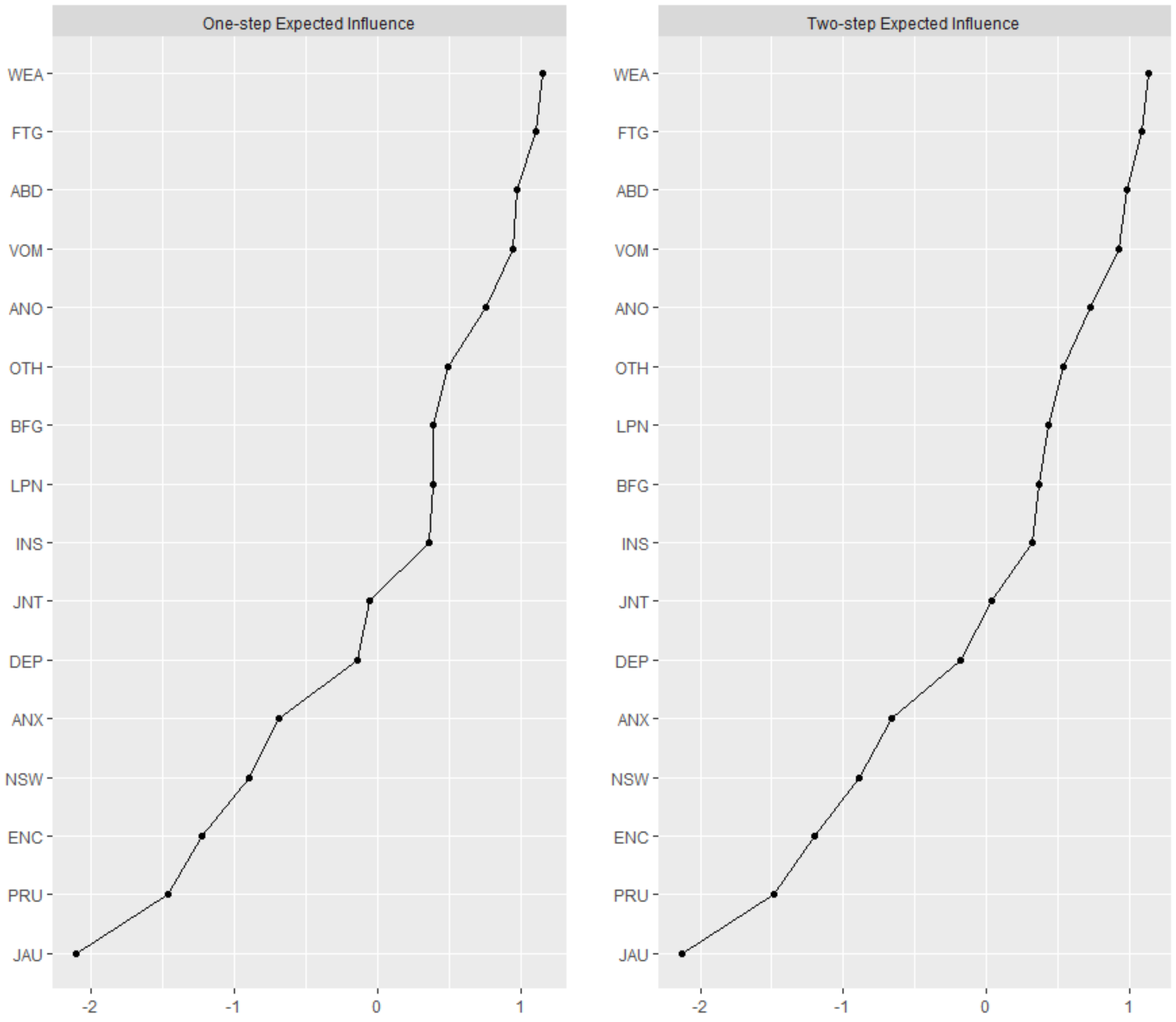

Supplementary Figure 3. Expected influence z-scores for LASSO regularized partial correlation network of 16 symptoms reported at their worst (n=958)

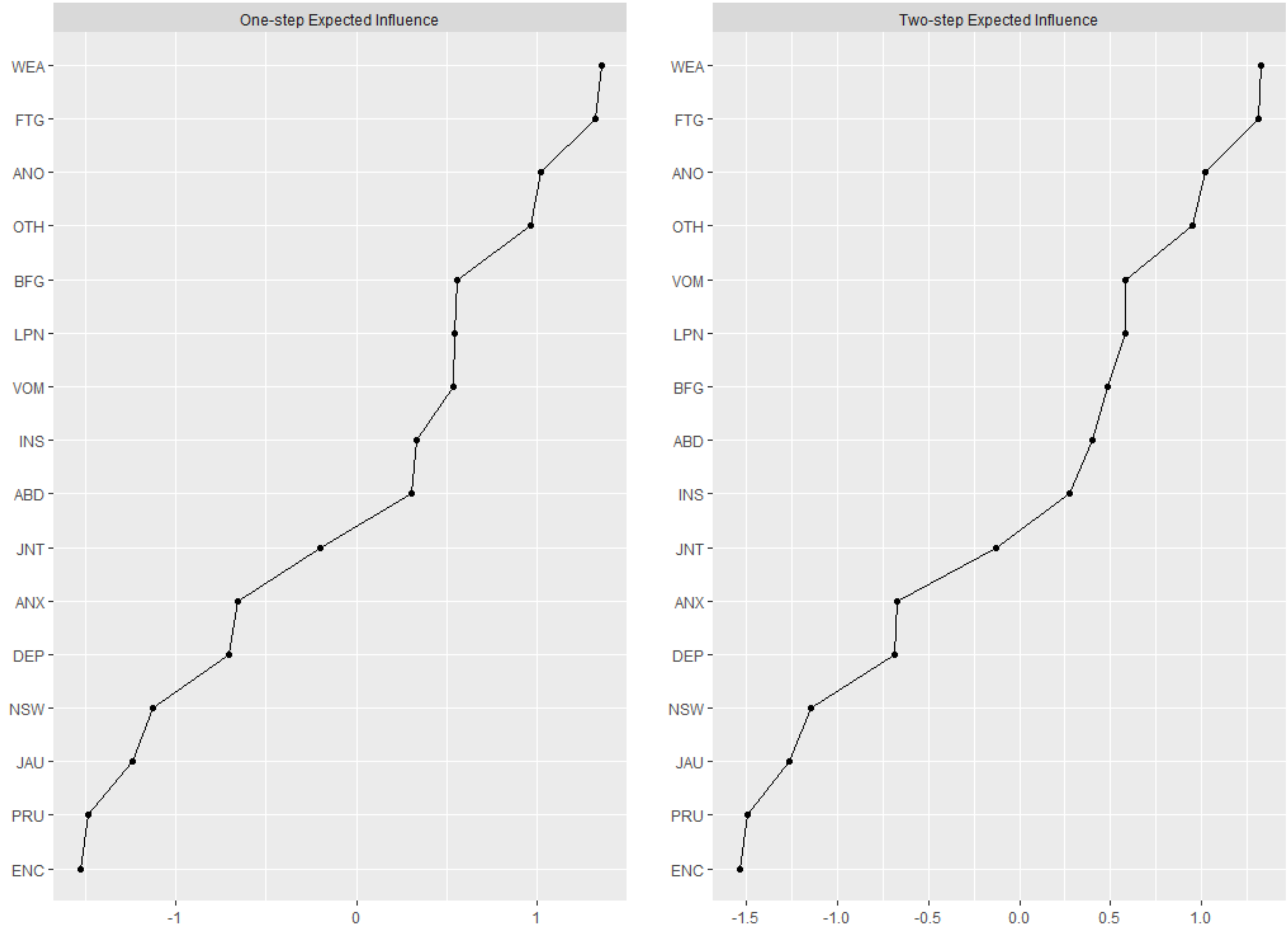

Supplementary Figure 4. One-step and two-step expected influence z-scores for LASSO regularized partial correlation network of 16 symptoms reported in the past 6 months among patients with IBD (n=530)

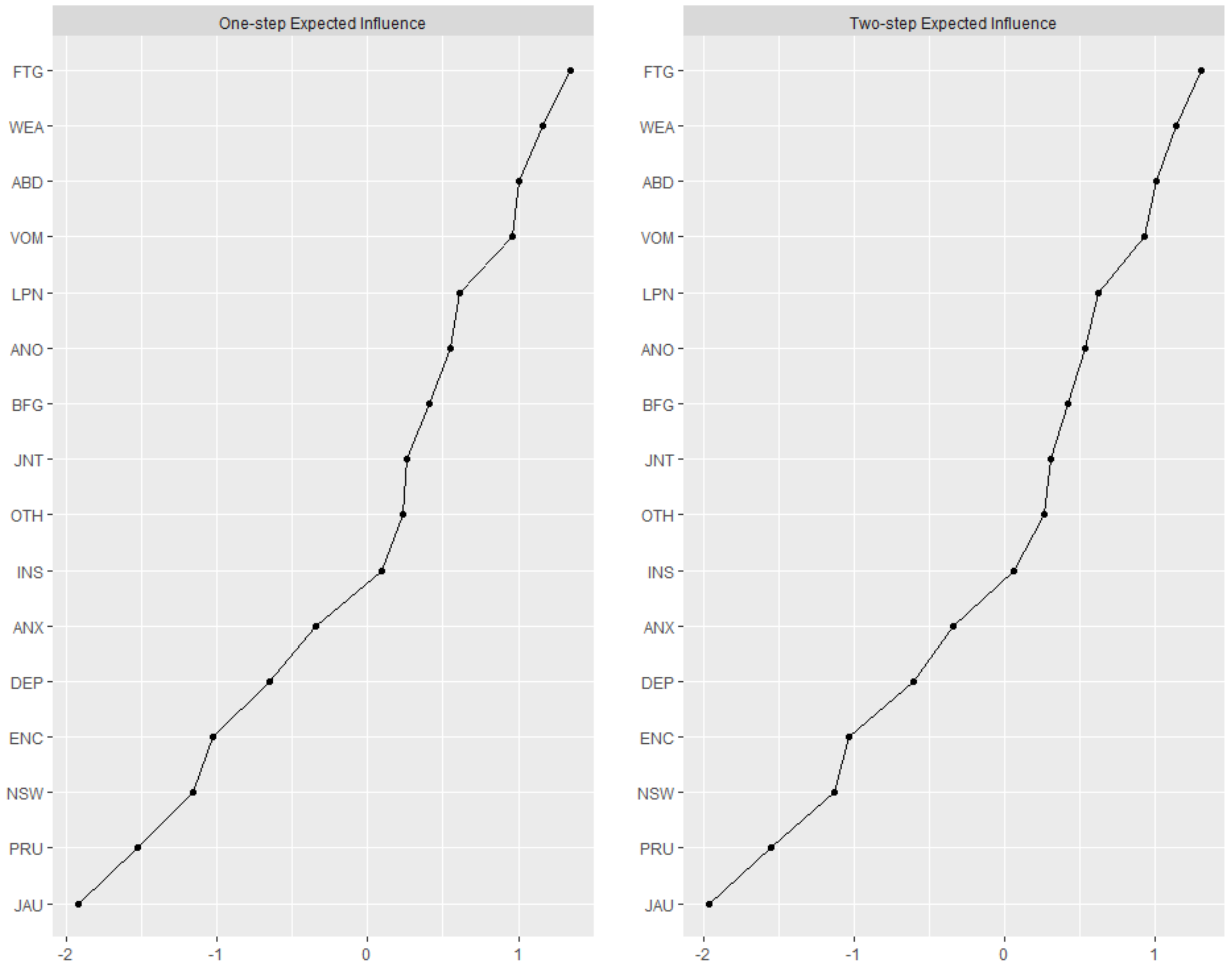

Supplementary Figure 5. One-step and two-step expected influence z-scores for LASSO regularized partial correlation network of 16 symptoms reported in the past 6 months among patients without IBD (n=234)

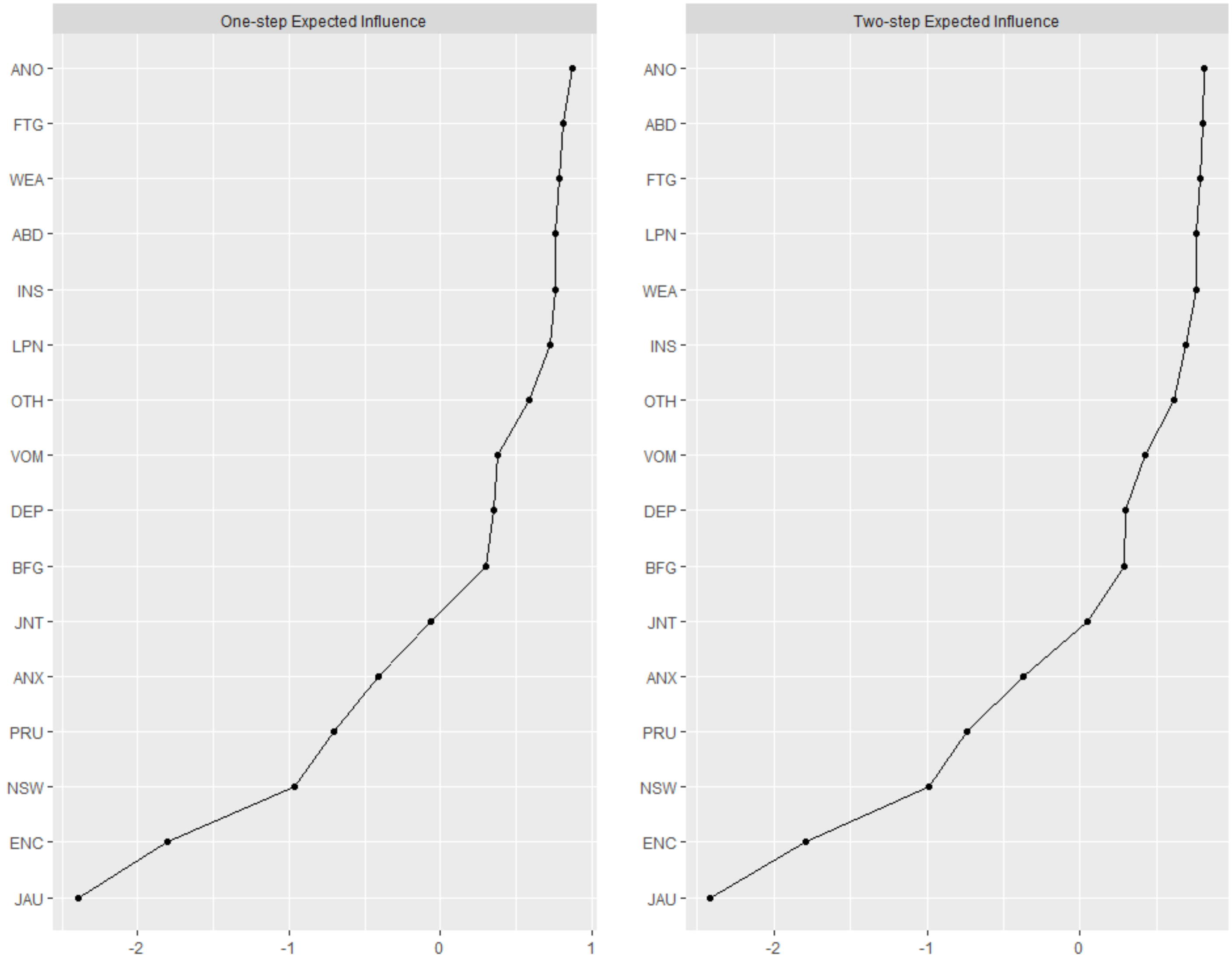

Supplement: Supplementary file 1 [file CJGH-2026-5291248-s002.pdf]
