# Supplementary material for: Symptom Network Analysis in Patients With Primary Sclerosing Cholangitis
Source: Can J Gastroenterol Hepatol. 2026 Oct 14;2026:5291248. doi: 10.1155/cjgh/5291248 (PMC13662822; doi:10.1155/cjgh/5291248)
Supplement: Supplementary file 2 [file CJGH-2026-5291248-s001.docx]

**Supplemental Table 1. Impact of Symptoms on How Patients with PSC Feel and Function**

| **Symptom** | **6 months***  **N=970** | **Current***  **N=970** | **Worst**  **N=970** |
| --- | --- | --- | --- |
| Fatigue  Never  Minor  Major | 138 (18)  325 (42)  312 (40) | 215 (28)  306 (39)  256 (33) | 95 (10)  257 (27)  616 (64) |
| Weakness  Never  Minor  Major | 375 (48)  288 (37)  111 (14) | 461 (59)  223 (29)  93 (12) | 311 (32)  384 (40)  274 (28) |
| Pruritus  Never  Minor  Major | 329 (43)  302 (39)  143 (18) | 460 (59)  208 (27)  108 (14) | 267 (28)  287 (30)  414 (43) |
| Anorexia  Never  Minor  Major | 411 (53)  299 (39)  65 (8) | 536 (69)  187 (24)  51 (7) | 350 (36)  376 (39)  243 (26) |
| Nausea/Vomiting  Never  Minor  Major | 400 (52)  284 (37)  90 (12) | 539 (69)  184 (24)  54 (7) | 396 (41)  328 (34)  244 (25) |
| Liver Pain  Never  Minor  Major | 301 (39)  352 (46)  122 (16) | 465 (60)  229 (30)  80 (10 | 271 (28)  412 (43)  286 (30) |
| Abdominal Pain  Never  Minor  Major | 291 (38)  361 (47)  123 (16) | 453 (58)  228 (29)  94 (12) | 250 (26)  414 (43)  304 (31) |
| Joint Pain  Never  Minor  Major | 382 (49)  284 (37)  107 (14) | 451 (58)  236 (30)  87 (11) | 406 (42)  377 (39)  184 (19) |
| Other Pain  Never  Minor  Major | 343 (49)  269 (35)  50 (6) | 528 (68)  203 (26)  42 (5) | 482 (50)  356 (37)  129 (13) |
| Insomnia  Never  Minor  Major | 272 (35)  295 (38)  207 (27) | 350 (45)  251 (32)  174 (22) | 271 (28)  331 (34)  366 (38) |
| Night Sweats  Never  Minor  Major | 484 (62)  242 (31)  49 (6) | 571 (73)  171 (22)  35 (5) | 464 (48)  367 (38)  138 (14) |
| Brain Fog  Never  Minor  Major | 378 (49)  284 (37)  112 (14) | 463 (60)  222 (29)  91 (12) | 370 (38)  378 (39)  219 (23) |
| Encephalopathy  Never  Minor  Major | 681 (88)  65 (8)  26 (3) | 701 (90)  51 (7)  23 (3) | 779 (81)  108 (11)  79 (8) |
| Anxiety  Never  Minor  Major | 313 (40)  323 (42)  139 (18) | 394 (49)  279 (36)  113 (15) | 328 (34)  387 (40)  254 (26) |
| Depression  Never  Minor  Major | 342 (44)  303 (39)  128 (17) | 431 (56)  249 (32)  96 (12) | 345 (36)  406 (42)  216 (22) |
| Jaundice  Never  Minor  Major | 613 (79)  126 (16)  33 (4) | 677 (88)  73 (9)  22 (3) | 519 (54)  241 (25)  206 (21) |

*Excluding transplanted patients

**Supplemental Table 2. Illustrative Quotations of Patients with PSC and their symptoms**

| **Symptoms** | **Illustrative Quotation** |
| --- | --- |
| Multi-symptom | “The PSC symptoms of severe fatigue, cirrhosis, vomiting, lack of appetite, stomach issues, sleeplessness and brain fog deeply affected my quality of life and my mental health.” |
|  | “I've been experiencing debilitating fatigue, bone, muscle pain, IBD, nausea, brain fog, memory loss, itching, vitamin deficiencies, and more, so many sick days I can no longer work." |
|  | “I had itching, fatigue, constant fever and chills, and extreme abdominal pain.” |
| Pain | “My pain is so unbearable and severe that I take it out on my loved ones….I isolate myself away from my friends. The pain which is chronic and doesn't improve with medication for me makes me feel trapped in this progressively diseased body…. This pain will always be a part of my life and it will never let me forget it. “ |
| Insomnia | “I'm awake out all hours of the night itching and many days, I'm so tired that after an eight hour nursing shift, I sit alone crying…There are even days when I'm not able to stand in the shower because I'm just too tired. I also have pain in my upper right quadrant and that pain is so bad. At night, I get really irritated at myself for not being able to cope with it. It feels almost like a shark biting the side of my liver radiating to my back and shoulders.” |
|  | “I am missing so much of life, and sleeping it away. And, I have no choice because if I push myself, I end up making myself sick.” |
| Fatigue | “The emotional toll of this disease is completely devastating. It's knowing you have so much potential in life, but you can't reach your goals or wishes because you're just too tired to get out of bed that day…..It's being trapped inside a brain and body that no longer work together.” |
|  | “PSC fatigue is like having a faulty battery that you put on a charger overnight. You never know if you're going to get a full charge, a half a charge or maybe a dead battery the next morning…..Fatigue causes headaches, severe nausea, lightheadedness just to name a few….Even after having nine hours of sleep, sometimes I wake up with a dead battery, even after a nap, a simple task like emptying the dishwasher, folding my laundry, it'll send me right back to bed.” |
|  | “Living with PSC and dealing with fatigue on a regular basis, it prevents me from working, from spending time with my friends and my family. And it prevents people from relying on me. “ |
|  | “They don't really understand that, it's not go, go, go all the time, you have to stop and rest….. I simply, physically cannot keep going sometimes.” |
|  | “Imagine yourself for a period of several years, when you wake up each morning, you do not feel rested despite having slept the night through. And then you need a midday nap, just to make it through to the evening. What a way to spend your late twenties and thirties dealing with this constant fatigue because of PSC” |
| Pruritus | “My primary symptom is unrelenting itching…. My itching has been my greatest disability….it consumes me throughout my entire day with itching from my scalp to the bottom of my feet. There is no spot on my body that doesn't crave attention. Sometimes it becomes almost unbearable. At night, when there are no distractions, my body unleashes an attack of itching. So significant that I end up tearing up scars previously made and leaving bloodstains on my pillows and sheets.” |
|  | “Itching slows me down each morning as it interrupts every segment of my normal routine getting out of bed, brushing my teeth, getting dressed are all delayed due to the itching.” |
|  | “Itching hurts….like someone is taking sand paper to my skin which feels inflamed and raw, the more I itch my skin, the more it hurts. As I remove my clothes to go to bed, my body feels superheated like I sat in the sun too long. My scalp is very painful….my eyes can hurt from the scratching.” |
|  | “This debilitating itch is merciless, all consuming, and overwhelming. It's everywhere and worst of all, my feet. I don't even notice when I'm itching anymore and I find myself rubbing my feet on anything, creating blisters. The pruritus increases in intensity and severity in the heat and at night. When I wake up, I've noticed that I've broken skin and started bleeding from itching subconsciously, creating scars. The itching results in a lack of sleep, making it difficult to function the following day.” |
|  | “Despite how minor the symptom of itching looks on paper, suffering from pruritus is beyond description.” |
| Brain Fog | “What you don't see is the amount of concentration it takes for me just to keep up with the conversation or when I stop in the middle of my sentences, because I can't remember what I was going to say next or when I slow my words. “ |
|  | “Brain fog is the best way to describe it…you have a word that you want to say, but you can't see through the fog, and actually grab it. You can't complete that sentence. You know you have the vocabulary to make it succinct, but you just don't have that ability to actually put it into words that you can say. It is reading a really good book, and the next day picking it up to start reading it again, and not remembering what was in that chapter you read the day before. “ |

**Supplementary Table 3. Symptoms with most and least positive influence in each network**

| **Network** | **Category** | **1-Step Expected Influence (EI1)** | **2-Step Expected Influence (EI2)** |
| --- | --- | --- | --- |
| **1: symptoms reported in past 6 months (n=764)** | Most positive influence | FTG: Fatigue  WEA: Weakness | FTG: Fatigue  WEA: Weakness |
|  | Least positive influence | JAU: Jaundice  ENC: Encephalopathy | JAU: Jaundice  ENC: Encephalopathy |
| **2: current symptoms (n=758)** | Most positive influence | WEA: Weakness  FTG: Fatigue | WEA: Weakness  FTG: Fatigue |
|  | Least positive influence | JAU: Jaundice  PRU: Pruritus | JAU: Jaundice  PRU: Pruritus |
| **3: symptoms reported at their worst (n=958)** | Most positive influence | WEA: Weakness  FTG: Fatigue | WEA: Weakness  FTG: Fatigue |
|  | Least positive influence | ENC: Encephalopathy  PRU: Pruritus | ENC: Encephalopathy  PRU: Pruritus |
| **4: symptoms reported in past 6 months among patients with IBD (n=530)** | Most positive influence | FTG: Fatigue  WEA: Weakness | FTG: Fatigue  WEA: Weakness |
|  | Least positive influence | JAU: Jaundice  PRU: Pruritus | JAU: Jaundice  PRU: Pruritus |
| **5: symptoms reported in past 6 months among patients without IBD (n=234)** | Most positive influence | ANO: Anorexia FTG: Fatigue | ANO: Anorexia ABD: Abdominal Pain |
|  | Least positive influence | JAU: Jaundice  ENC: Encephalopathy | JAU: Jaundice  ENC: Encephalopathy |

**Supplementary Table 4. Community Summaries across Network Analyses by Symptom**

|  | Network 1 | Network 2 | Network 3 | Network 4 | Network 5 |
| --- | --- | --- | --- | --- | --- |
| Joint Pain | 1 | 1 | 6 | 1 | 2 |
| Other Pain | 1 | 1 | 6 | 1 | 2 |
| Brain Fog | 1 | 4 | 2 | 2 | 2 |
| Encephalopathy | 1 | 4 | 2 | 2 | 2 |
| Weakness | 1 | 1 | 3 | 1 | 2 |
| Fatigue | 1 | 4 | 3 | 1 | 3 |
| Pruritus | 4 | 4 | 5 | 6 | 3 |
| Jaundice | 4 | 4 | 5 | 6 | 3 |
| Insomnia | 4 | 4 | 5 | 3 | 3 |
| Night Sweats | 4 | 4 | 5 | 3 | 4 |
| Anxiety | 2 | 2 | 1 | 5 | 4 |
| Depression | 2 | 2 | 1 | 5 | 4 |
| Anorexia | 3 | 3 | 4 | 4 | 1 |
| Nausea/vomiting | 3 | 3 | 4 | 4 | 1 |
| Liver Pain | 3 | 3 | 4 | 4 | 1 |
| Abdominal Pain | 3 | 3 | 4 | 4 | 1 |
